# Report on the Teeth of a Mound-Builder

**Published:** 1884-02

**Authors:** E. G. Betty


### Report on the Teeth of a Mound-Builder.

BY E. G. BETTY, D.D.S.

Read before the Natural History Society of Cincinnati, January 8, 1884.

Walter A. Dun, M. D., of this city, read an interesting and exhaustive paper upon the excavation of a mound situated on Deer Creek, Ross County, Ohio, and about nine miles from Chillicothe. The excavation was made in June, 1876, in company with Mr. Scott Cunningham, a resident of the above town. The mound is situated on a bluff, and is covered with a dense native forest. No evidences of fire were found, proving that the mound was not used as a station from which signals were made by large fires, but used solely as a place of sepulchre. The measurements, as near as could be determined, were: circumference, 361 feet and a few inches; diameter, 115 feet; height, 33 feet. "Calculations from these figures show that the area covered by the base of the mound, was 10,384½ square feet, and the entire amount of earth used in its construction was 114,229½ cubic feet," all of which was carried from a distance with great labor. The excavation proceeded slowly, as the entire depth reached by the shaft was 35 feet from the apex of the mound to the base line upon which was found the remains of a male skeleton, together with some relics, as flint arrow-heads, shells in the form of wampum, brought from the shores of the Gulf of Mexico where only they are found, and last, but not least, an octagonal stone about two inches in diameter and half an inch thick; this stone being regarded as evidence that the mound-builders were more or less acquainted with the science of geometry.

The coffin or sarcophagus was composed of oak logs, undressed, but long since decayed, and measured eight feet long from east to west, and five and one-half feet wide from north to south, and four feet high, the measurements being taken from out to out from the molds left in the soil.

The crypt was a vault about ten feet high at its apex from the base line of the mound, and on the roof or ceiling of which was found a fungi hanging pendant, like some kind of moss, and from ten to fifteen inches in length of fiber. The skeleton was six and one-half feet in length, of very massive structure, and in the recent state must have been very muscular. About two-thirds of the skull were recovered, a portion of a femur and tibia, and a few fragments, the identity of which can hardly be determined. The skull-bones were boiled in glue for some time to give them strength by filling up the interstices with animal matter and then varnished.

The doctor proceeded very much in detail, giving, as he went along, all the points, and wherein they cöincided or differed with the authorities. There have been found only six or eight, possibly ten skulls, of undoubted mound-builders, the one here described having all the characteristics of those well vouched for.

The paper will be published in the transactions of the society, and any one interested can, no doubt, procure a copy by writing to the Secretary.

The point concerning the dentists most, is the description of the jaws and teeth made by the writer at the request of Dr. Dun, and which, by the way, is the *first* report upon the teeth of a genuine mound-builder that has ever been made.

This report was read at the conclusion of the account of the "find," and it brought out points that proved the correctness of the doctor's theory concerning the race of the individual, etc., though it had been made solely upon the specimen as it is, and without consultation with Dr. Dun :

THE JAWS

Are both massive; particularly so, the inferior maxilla; in size they are somewhat larger and seemingly more compact than the average jaws seen at the present day.

THE INFERIOR MAXILLARY.

This bone is minus the posterior portion on the right side, including the angle, ramus, and condyle, being broken off, beginning below at a point just anterior to the second molar, and running backwards and upwards to a point about one line back of the third molar, and one-half inch anterior to the posterior mental foramen.

The ridges for the attachment of the muscles are very prominent and well defined, especially that for the masseter.

The genial tubercles are not definitely marked, the four merging together in one large roughened surface or patch, affording a secure attachment for muscles operating the jaw. The entire jaw, or at least the remaining portion of it, is so strong and heavy that it suggests the possession of very powerful muscles to have given it the necessary force required in mastication.

It was also well nourished, as the many minute foramina, for the entrance and exit of bloodvessels to the interior and substance of the bone, are very numerous.

A prominent feature of the jaw is the unusual distance from the superior margin of the alveoli to the lower border of the bone, extending from the second bi-cuspid on one side to that on the other.

This feature is one characteristic of those skulls found in several parts of the Mississippi Valley, and described by Schoolcraft and others.

Of the teeth in this jaw, fifteen have been erupted, fourteen remaining; the fifteenth (the second molar on the left side) is missing, evidently having been lost during the excavation of the mound.* This is very probable from the fact that the septum between the two roots is well marked on the lingual side, and the alveolus is of such a depth as to preclude the possibility of its loss during life, for, in that event, it would have been filled up with porous bony tissue, and with a more or less compact surface. Also, the great abrasion of the superior second and third molars, is conclusive evidence that it existed up to the time of death.

The third molar of the left side is not present, the germ being entirely absent, so, of course, it was never erupted, the ridge in that locality presenting a smooth, compact surface; besides this, there is a total absence of abrasion on the posterior half of the superior third molar on the same side.

The interesting point in connection with the non-eruption of this third molar, and also that of the upper right side, is the fact

* This tooth was not lost at all, but misplaced, having been found in its proper place in the jaw. This I did not know until Dr. Dun read the report.—[E. G. B.

that there is a seemingly well-substantiated theory that the civilized races are gradually but surely losing the third molar altogether by reason of its falling into disuse, the little care taken of it, the systematic extraction of it, etc.

In the specimen under consideration, two of the four third molars having not erupted, would seem to negative that theory, though their absence may only be peculiar to that individual; still the fact exists.

All of the teeth in this jaw are of very massive construction, solid, square, and well developed in every particular; the dentine dense and resistant, the enamel very thick, and of prime quality.

Furthermore, they do not show the slightest trace of dental caries of any variety, the only disease having been an alveolar abscess upon the roots of the first molar on the left side, that destroyed the buccal wall of the socket, down to the apices of the roots.

The curves of the arch are perfectly even and symmetrical, such as seen in normal mouths of the present time; no irregularity in the position of the teeth whatever.

With the exception of the third molar on the right side, all the teeth of this jaw are more or less worn down by mechanical abrasion, the natural result of a full and long-continued use of the jaws in the mastication of food, especially if it be coarse and difficult of comminution, as that of the mound-builders undoubtedly was, though it is the opinion of some that they subsisted mainly by the chase.

The four incisors are worn down more than one-half their length, at any rate; the cuspids nearly or quite one-half; the cusps of the bicuspids are ground off just to the dentine; the first and second molars of the right side are worn away about one-half, while the first molar on the left side is, on the posterior buccal side, worn pretty nearly to the gum line, which, no doubt, accounts for the abscess at its roots, there being no trace of caries.

All of the teeth at their necks have rings of salivary calculus, both inside and out, but not in unusual quantity. The septa between the teeth, especially those of the ten anterior teeth, are well defined and without loss of substance, standing in their original completeness, evidencing an absence of disease of the gums, as shown by the outlines of the deposits.

THE SUPERIOR MAXILLARY.

This jaw, like the generality of those of the mound builders, is large and massive, the palatine arch being broad and deep; the alveolar ridge is high and well developed, affording a very firm attachment for the large teeth, being very prominent in front of the six anterior teeth. The palate is exceedingly rough and deeply indented at the sides for the passage of the posterior palatine vessels, which make their entrance through very large foramina.

It is also pierced by a very large anterior palatine foramen, its diameter about that of a goose quill. The very rough condition of the anterior portion of the palate would seem to indicate that, in the recent state, the rugae must have been well marked and quite prominent. Of the teeth remaining there are fifteen, the absent one being the third molar of the right side, there being no evidence of its having been erupted.

On the whole, the teeth of this jaw, though of about the same quality and size as those of the lower jaw, do not present so good an appearance. Yet the enamel upon them is very thick and of excellent structure, fully as good originally as that upon the teeth of the lower jaw.

They are very much worn down by mechanical abrasion; the four incisors clear to what must have been the gum line on the lingual sides, and to within half a line of it on the labial surfaces.

The cuspids are also very much worn away, and pretty close to the gum, both being "cupped," or hollowed out upon the grinding surface, particularly the left one, the pulp-chamber having been exposed, as is also the case with the left lateral incisor and second right bicuspid.

The four bicuspids are worn away, nearly if not quite one-half the original length of their crowns.

It is the second and third molars of both sides, however, that exhibit the most extensive abrasion.

On their lingual sides they are gone clear to the gum and above their necks; so great is the cupping that it can only be accounted for by the action of solvents in the process known as chemical abrasion. The grinding surfaces are smooth and dense, showing the dentine to have been well organized.

Of the first right molar, the entire diameter of the pulp chamber is exposed, the subsequent death of the pulp, resulting in an abscess at the roots that destroyed the outer plate of the alveolus.

Abscesses also existed at the roots of the same tooth on the opposite side, as is the case, too, with the first bicuspid and lateral incisor; and possibly, also, of the cuspid between them, also on the left side.

The first bicuspid of this side was evidently broken off during life, and at the margin of the gum, no doubt remaining for a long time as a mere root, though a painful one.

The abscess on the left first molar made two large openings into the antrum.

None of the teeth in this jaw exhibit any traces of dental caries whatever, the abscesses having been caused by the irritation and death of the pulps, incident upon the extensive abrasions. There is some absorption of the process around two or three of the molars, no doubt due to accretions of calculus. With these exceptions, the deposits of tartar are insignificant, though present upon all the teeth; and, judging by the well-defined septa between them, the soft parts were in a good and healthy condition.

When occluded, the articulation of the teeth in both jaws is as nearly normal as usually seen in mouths now-a-days, taken as they come. The superior anterior teeth overlap the inferior, as they should; the upper molars and bicuspids, instead of slightly overlapping the lower, strike square upon each other.

The median line in this specimen does not pass directly between the central incisors of both jaws, but bisects the lower teeth through the center of the right central incisor, thus placing the left half of the teeth about one line to that side of the center.

In profile, the teeth are on the perpendicular, the eminence at the symphysis making the point of the chin slightly prominent, the whole having the general form of the Caucasian.

This description of the jaws and teeth of the specimen under consideration is made minute for the reason that it is as nearly perfect a specimen as can be found, and is typical of what the normal mouth of the mound-builder was.

